# Observability Analysis of a MEMS INS/GPS Integration System with Gyroscope G-Sensitivity Errors

**DOI:** 10.3390/s140916003

**Published:** 2014-08-28

**Authors:** Chen Fan, Xiaoping Hu, Xiaofeng He, Kanghua Tang, Bing Luo

**Affiliations:** College of Mechatronics and Automation, National University of Defense Technology, Changsha 410073, China; E-Mails: ff_chenlin@yeah.net (C.F.); hexiaofeng@nudt.edu.cn (X.H.); tt_kanghua@hotmail.com (K.T.); ruobing@nudt.edu.cn (B.L.)

**Keywords:** MEMS INS/GPS integration system, observability analysis, gyro g-sensitivity errors, linear acceleration effect on gyros

## Abstract

Gyroscopes based on micro-electromechanical system (MEMS) technology suffer in high-dynamic applications due to obvious g-sensitivity errors. These errors can induce large biases in the gyroscope, which can directly affect the accuracy of attitude estimation in the integration of the inertial navigation system (INS) and the Global Positioning System (GPS). The observability determines the existence of solutions for compensating them. In this paper, we investigate the observability of the INS/GPS system with consideration of the g-sensitivity errors. In terms of two types of g-sensitivity coefficients matrix, we add them as estimated states to the Kalman filter and analyze the observability of three or nine elements of the coefficient matrix respectively. A global observable condition of the system is presented and validated. Experimental results indicate that all the estimated states, which include position, velocity, attitude, gyro and accelerometer bias, and g-sensitivity coefficients, could be made observable by maneuvering based on the conditions. Compared with the integration system without compensation for the g-sensitivity errors, the attitude accuracy is raised obviously.

## 1. Introduction

The Global Positioning System (GPS) and Inertial Navigation System (INS) are used for obtaining information about position, velocity, and attitude. The integrated INS and GPS navigation system can take advantage of the features of both systems to enhance the performance of a navigation system. Recently, with the development of micro-electromechanical system (MEMS) technology, more and more inertial sensors based on MEMS have become feasible options to construct integrated INS/GPS systems [1]. Those low-cost and small sized inertial sensors meet the demands of high precision and reliability, and the applications of INS/GPS integrated systems can be expanded to many high-dynamic fields, such as guided weapons and unmanned aerial vehicles [2]. However, due to mass imbalance, most MEMS gyroscopes exhibit a sensitivity to line acceleration, commonly known as the g-sensitivity error, which causes further errors [3,4]. As shown in [5], the g-sensitivity error is one of the most important performance parameters of gyroscopes in high dynamic circumstances. These errors can induce large biases in gyroscope, also named g-dependent biases [4], which directly affect the accuracy of attitude estimation in the integrated system, and with the increase of line acceleration, the accuracy of attitude estimation becomes lower. Although the high dynamic period may be short, the accumulation of errors over time cannot be neglected [6]. Because the errors relate to the acceleration, the g-dependent biases would increase with any increase of the acceleration. Accordingly, in order to achieve optimal accuracy, it is necessary to focus on compensating for the gyroscope g-sensitivity errors in the integrated system.

Currently, there is not enough literature data to determine gyroscope g-sensitivity errors or design correction methods, and its observability in integrated MEMS INS/GPS navigation is rarely compared to other related gyroscope errors. The g-sensitivity error is mentioned in [4,7–9]. However, models, error analysis and methods of estimation are not given. In order to measure the Earth's rotation rate, the g-sensitivity error is built as a linear model and estimated in a calibration process [10]. Even though a Kalman filter is used for the mitigation of g-sensitivity errors in [6], whereby the g-sensitivity coefficients are adding as estimated states, the benefit is limited and affected by the observability of the coefficients. It is worth mentioning that the works to estimate the g-sensitivity error are limited and need to be directed by the observability analysis.

Observability is an important aspect of the state estimation problem in the integration system as it determines the existence and nature of solutions [11]. For the nonlinear INS/GPS system, the observability analysis was usually performed using the corresponding linearized models. However, the linearization is more implicit when considering g-sensitivity errors. Since the linearization implies that the observability can only locally characterize the properties of the original nonlinear system, e.g., local observability [12], the results could be mainly theoretical and far away from engineering applications. Recently, a global concept was used to analyze the observability of a nonlinear system, and some significant conclusions were obtained [11,13]. The global concept describes the ability to estimate the states in the whole time span whereas the local observability concepts deal with the ability to distinguish the states from their neighbors in a small time interval or instantaneously [13]. The global observability analysis starts directly from the basic observability definition and can guide us on how to achieve the state estimate by resorting to maneuvers, because maneuvers could improve the observability of states in the integration system [14]. This approach can also be used for analyzing the observability of the integrated system when it is applied to other applications, such as land vehicles [11]. This paper provides a sufficient condition of observability which is based on the global observability analysis approach. We adopt a linear g-sensitivity model and add its coefficients as states to be estimated in a Kalman filter. In terms of two types of the g-sensitivity coefficients matrix, we analyze the observability of three or nine elements of the matrix, respectively. Based on the observable conditions, the type of aircraft maneuvers is presented to correct the gyroscope g-sensitivity errors in motion. A simulation is performed to support the theoretic results. It is shown that when designing the reasonable maneuvering conditions, all the error states of system are observable and correctly estimated.

This paper is organized as follows: Section 2 discusses the measurement errors model of a MEMS gyroscope. Then a nonlinear dynamic model of a MEMS INS/GPS system is presented, and the Kalman filtering with g-sensitivity errors is designed. Section 3 is devoted to an observability analysis for the all error states of the model, mainly discussing the observability properties of the g-sensitivity coefficients. Section 4 presents the simulation and results. Finally, conclusions are provided in Section 5.

## 2. Model Description

In this paper, we first give the measurement equation with MEMS gyro g-sensitivity error. Then the nonlinear dynamic model of integration system is introduced and discussed. After that, we design a Kalman filter with the g-sensitivity coefficients to realize g-sensitivity error compensation.

### 2.1. Measurement Equation with Gyro g-Sensitivity Errors

Within the scope of this paper, we address the gyro bias, g-sensitivity error and noise. Other error sources, such as scale factor and cross-coupling, have been accurately compensated through factory calibration and allow for increased focus on the g-sensitivity error. Since the g-sensitivity errors are due due to mass imbalances, and are related to the acceleration in high-dynamic applications, they could induce large biases in the gyroscope that increase with acceleration. Therefore, the gyro measurement equation can be expressed as:

(1) $${\tilde{\text{ω}}}_{ib}^{b}={\text{ω}}_{ib}^{b}+{\text{b}}_{g}+\text{δ}{\text{ω}}_{ib}^{b}+{\text{η}}_{g}$$

where ${\tilde{\text{ω}}}_{ib}^{b}$ is the angular rate measurement of the gyroscope, ${\tilde{\text{ω}}}_{ib}^{b}$ is the body angular rate with respect to the *b*-frame, ***b****_g_* are the gyro biases (also named g-independent biases [4]), $\text{δ}{\tilde{\text{ω}}}_{ib}^{b}$ are the gyro g-sensitivity errors, ***η****_g_* is the noise of the gyro measurements.

According to the present in [6], the model of g-sensitivity errors can be expressed as:

(2) $$\text{δ}{\text{ω}}_{ib}^{b}=G{\text{f}}_{ib}^{b}$$

where *G* is a 3 × 3 matrix encompassing the g-sensitivity coefficients, ${\text{f}}_{ib}^{b}$ is the specific force measured by accelerometers expressed in the *b*-frame. In general, the g-sensitivity coefficient matrix is separated into three components, each a 3 × 3 matrix:

(3) $$G=G_{o}+G_{s}+G_{d}$$

In Equation (3), the g-sensitivity coefficients in *G_o_* remain unchanged and are typically estimated in a factory calibration. The *G_s_* is the turn on g-sensitivity coefficients, which remains constant but varies each time the gyro is powered on. The third aspect is the dynamic in-run g-sensitivity coefficients. In this paper, *G_o_* is primitively neglected due to the factory calibration, allowing our focus on the *G_s_* and *G_d_*. Because the *G_s_* and *G_d_* are hard to estimate separately, so they are referred to as the *G* matrix and named the residual g-sensitivity errors.

Traditionally, it can be expressed as the three-element diagonal matrix or nine-element matrix:

(4) $$G=\left[\begin{array}{ccc} G_{11} & 0 & 0\\ 0 & G_{22} & 0\\ 0 & 0 & G_{33} \end{array}\right]$$

or:

(5) $$G=\left[\begin{array}{ccc} G_{11} & G_{12} & G_{13}\\ G_{21} & G_{22} & G_{23}\\ G_{31} & G_{32} & G_{33} \end{array}\right]$$

### 2.2. Nonlinear Dynamic Model

The dynamic equations for a strap down INS are given by [15] in the Earth-centered Earth-fixed (ECEF) frame, which is denoted by *e*-frame. The body frame is denoted by *b*-frame with the axes pointing forward, right, and up; the inertial frame is denoted by *i*-frame; and the body-fixed local level frame is denoted by *n*-frame with the axes pointing to north, east, and down, respectively.

Converting the dynamic equations with respect to Equations (1) and (2), that is:

(6) $${\dot{\text{r}}}^{e}={\text{v}}^{e}$$

(7) $${\dot{\text{v}}}^{e}={\text{C}}_{b}^{e}\left({\text{f}}_{ib}^{b}-{\text{b}}_{a}\right)-2{\text{ω}}_{ie}^{e}\times {\text{v}}^{e}+{\text{g}}^{e}$$

(8) $${\dot{\text{C}}}_{b}^{e}={\text{C}}_{b}^{e}\left({\text{ω}}_{eb}^{b}\times \right)$$

(9) $${\text{ω}}_{eb}^{b}={\text{ω}}_{ib}^{b}-{\text{b}}_{g}-G{\text{f}}_{ib}^{b}-{\text{C}}_{e}^{b}{\text{ω}}_{ie}^{e}$$

where ${\dot{\text{r}}}^{e}$ and ${\dot{\text{v}}}^{e}$ are the position and velocity of INS in the *e*-frame, ${\text{C}}_{b}^{e}$ is the body attitude matrix with respect to the *e*-frame, ***b****_a_* is the accelerometer bias, ${\text{ω}}_{ie}^{e}$ is the Earth's rotation rate expressed in the *e*-frame, ***g**^e^* is the gravity vector in the e-frame, ${\text{ω}}_{eb}^{b}$ is the body angular rate with respect to the *e*-frame and expressed in the *b*-frame, $\left({\text{ω}}_{eb}^{b}\times \right)$ is the skew-symmetric matrix of ${\text{ω}}_{eb}^{b}$. In this paper, the gyro bias ***b****_g_* and the accelerometer bias ***b****_a_* are taken as random constants for simplicity, *i.e.*:

(10) $${\dot{\text{b}}}_{g}=0$$

(11) $${\dot{\text{b}}}_{a}=0$$

According to the conclusions in [6], the best model for the g-sensitivity coefficients matrix *G* is the random constant, *i.e.*:

(12) $$\dot{G}=0$$

Supposing that the position and velocity can be measured by a single-antenna GPS receiver without errors, the measurement equation is described as:

(13) $${\text{z}}_{1}={\text{r}}^{e}$$

(14) $${\text{z}}_{2}={\text{v}}^{e}$$

### 2.3. Kalman Filtering with g-Sensitivity Coefficient

In this paper, the type of INS/GPS integrated navigation scheme is a loosely coupled integration, the bases of which are given in [4]. We add the gyro g-sensitivity coefficients as states to the Kalman filter. Due to the two different types of the g-sensitivity coefficients matrix, there are both three-state addition and nine-state addition to consider. Considering other states, namely three states for the position, three states for the velocity, three states for attitude, six states for gyro and accelerometer biases, the filter consists of 18 or 24 states.

The attitude error equation has been given in [4]. Since the g-sensitivity errors could induce biases in the gyroscope, we rewrite it with gyro g-sensitivity errors, and thus we have:

(15) $$\dot{\text{ɛ}}=-{\text{ω}}_{ie}^{e}\text{ɛ}+{\text{C}}_{b}^{e}{\text{b}}_{g}+{\text{C}}_{b}^{e}G{\text{f}}_{ib}^{b}$$

In general, the g-sensitivity errors are overlooked in the attitude error equation. Therefore, the Kalman filter model is not suitable for practical situations, especially in high-dynamic applications, and attitude estimation would contain the g-sensitivity errors. In other words, those errors could directly affect the accuracy of attitude estimation if not compensated. For the three-element matrix *G*, we rewrite Equation (2) using Equation (4) as:

(16) $$\delta {\text{ω}}_{ib}^{b}=F_{1}^{b}G_{1}$$

where:

(17) $$F_{1}^{b}=\left[\begin{array}{ccc} f_{\text{ibx}}^{b} & 0 & 0\\ 0 & f_{\text{iby}}^{b} & 0\\ 0 & 0 & f_{\text{ibz}}^{b} \end{array}\right]$$

(18) $$G_{1}={\left[\begin{array}{ccc} G_{11} & G_{22} & G_{33} \end{array}\right]}^{T}$$

Substituting Equation (16) into Equation (15):

(19) $$\dot{\text{ɛ}}=-{\text{ω}}_{ie}^{e}\text{ɛ}+{\text{C}}_{b}^{e}{\text{b}}_{g}+{\text{C}}_{b}^{e}F_{1}^{b}G_{1}$$

Then the 18-state vector can be expressed as follows:

(20) $$\text{X}=\left[\begin{array}{cccccc} \delta \text{r} & \delta \text{v} & \text{ɛ} & {\text{b}}_{g} & {\text{b}}_{a} & G_{1} \end{array}\right]$$

Analogously, we combine Equations (2) and (5), that is:

(21) $$\dot{\text{ɛ}}=-{\text{ω}}_{ie}^{e}\text{ɛ}+{\text{C}}_{b}^{e}{\text{b}}_{g}+{\text{C}}_{b}^{e}\left(F_{2}^{b}G_{2}+F_{3}^{b}G_{3}+F_{4}^{b}G_{4}\right)$$

where:

(22) $$\begin{array}{c} F_{2}^{b}={\left[\begin{array}{ccc} {\text{f}}_{ib}^{b} & 0 & 0 \end{array}\right]}^{T}\\ F_{3}^{b}={\left[\begin{array}{ccc} 0 & {\text{f}}_{ib}^{b} & 0 \end{array}\right]}^{T}\\ F_{4}^{b}={\left[\begin{array}{ccc} 0 & 0 & {\text{f}}_{ib}^{b} \end{array}\right]}^{T} \end{array}$$

(23) $$\begin{array}{c} G_{2}={\left[\begin{array}{ccc} G_{11} & G_{12} & G_{13} \end{array}\right]}^{T}\\ G_{3}={\left[\begin{array}{ccc} G_{21} & G_{22} & G_{23} \end{array}\right]}^{T}\\ G_{4}={\left[\begin{array}{ccc} G_{31} & G_{32} & G_{33} \end{array}\right]}^{T} \end{array}$$

Then the 24-state vector can be expressed as follows:

(24) $$\text{X}=\left[\begin{array}{cccccccc} \delta \text{r} & \delta \text{v} & \text{ɛ} & {\text{b}}_{g} & {\text{b}}_{a} & G_{2} & G_{3} & G_{4} \end{array}\right]$$

To keep the paper reasonably concise, the detailed state equation and observation of filtering are not presented, as those equations can be easily obtained from [4].

## 3. Observability Analysis with g-Sensitivity Errors

For the INS/GPS system with gyro g-sensitivity errors, the states to be estimated comprise the position, velocity, attitude, gyro and accelerometer bias, and g-sensitivity coefficients. The knowledge available includes the specific force measured by accelerometers, the body angular rate measured by gyros, the position and velocity measured by an accurate single-antenna GPS receiver. According to the observability definition in [16], if the initial states can be uniquely solved given the measurements in a finite-time interval, then the system is proved to be observable. Before proceeding, a lemma necessary in the analysis are given below:

Lemma [17]: For any two linearly independent vectors, if their coordinates in two arbitrary frames are given, then the attitude matrix between the two frames can uniquely be determined.

Since the position and velocity can be measured by a single-antenna GPS receiver without errors, the ***r****^e^* and ***v****^e^* are known at any time, *i.e.*, ***r****^e^* and ***v****^e^* are observable. Supposing that the fight path is a straight line, and the body attitude does not change, *i.e.*, ${\dot{\text{C}}}_{b}^{e}=0$. Taking the derivative on both sides of Equation (14) and using Equation (7), we have:

(25) $${\dot{\text{z}}}_{2}={\text{C}}_{b}^{e}\left({\text{f}}_{ib}^{b}-{\text{b}}_{a}\right)-2{\text{ω}}_{ie}^{e}\times {\text{z}}_{2}+{\text{g}}^{e}$$

Taking the derivative again on both sides yields:

(26) $${\text{C}}_{b}^{e}{\dot{\text{f}}}_{ib}^{b}={\ddot{\text{z}}}_{2}+2{\text{ω}}_{ie}^{e}\times {\dot{\text{z}}}_{2}-{\dot{\text{g}}}^{e}$$

Since the vector ${\text{ω}}_{ie}^{e}$ is the Earth's rotation rate, and the position and velocity are directly measured, so that the vector ***z****_2_*, gravity vector and their all-order derivatives are known (the errors due to GPS receiver measurement are negligible), respectively. Hence, the items on the right side of Equation (26) are already known. According to the Lemma, the attitude matrix ${\text{C}}_{b}^{e}$ can uniquely be determined on the condition that there exist two linearly independent ${\dot{\text{f}}}_{ib}^{b}\left(t_{1}\right)$ and ${\dot{\text{f}}}_{ib}^{b}\left(t_{2}\right)$ for *t*_1_ ≠ *t*_2_. Since all the terms in Equation (25) except ***b****_a_* are known, the accelerometer bias ***b****_a_* can uniquely be determined.

As the attitude does not change in a straight line path, we also have ${\text{ω}}_{eb}^{e}=0$. Taking the derivative on both sides of Equation (9) and using Equation (11) and Equation (12) yields:

(27) $$G{\dot{\text{f}}}_{ib}^{b}={\dot{\text{ω}}}_{ib}^{b}$$

Because the g-sensitivity coefficients matrix can be expressed as two forms in Equations (4) and (5), and their rules of observation herein are different, we discuss them separately. When *G* is a diagonal matrix, we substitute it into Equation (27), that is:

(28) $$\left[\begin{array}{ccc} G_{11} & 0 & 0\\ 0 & G_{22} & 0\\ 0 & 0 & G_{33} \end{array}\right]\;\left[\begin{array}{c} {\dot{f}}_{\text{ibx}}^{b}\\ {\dot{f}}_{\text{iby}}^{b}\\ {\dot{f}}_{\text{ibz}}^{b} \end{array}\right]=\left[\begin{array}{c} {\dot{\omega }}_{\text{ibx}}^{b}\\ {\dot{\omega }}_{\text{iby}}^{b}\\ {\dot{\omega }}_{\text{ibz}}^{b} \end{array}\right]$$

Noticing that the rank of *G* is 3, we know that if there are more than one path segments with ${\dot{\text{C}}}_{b}^{e}\neq 0$ on which each element of ${\dot{\text{f}}}_{ib}^{b}$ exist non-zero time, the diagonal matrix can be uniquely determined, *i.e.*, the g-sensitivity coefficients matrix is observable.

When *G* is a nine-element matrix, rewriting Equation (27) in components, that is:

(29) $$\left[\begin{array}{ccc} G_{11} & G_{12} & G_{13}\\ G_{21} & G_{22} & G_{23}\\ G_{31} & G_{32} & G_{33} \end{array}\right]\;\left[\begin{array}{c} {\dot{f}}_{\text{ibx}}^{b}\\ {\dot{f}}_{\text{iby}}^{b}\\ {\dot{f}}_{\text{ibz}}^{b} \end{array}\right]=\left[\begin{array}{c} {\dot{\omega }}_{\text{ibx}}^{b}\\ {\dot{\omega }}_{\text{iby}}^{b}\\ {\dot{\omega }}_{\text{ibz}}^{b} \end{array}\right]$$

where ${\dot{\text{f}}}_{ib}^{b}$ and ${\dot{\text{ω}}}_{ib}^{b}$ are expressed as components in *b*-frame. There are nine unknown elements in the equation. If there are three different-time measurement of ${\text{f}}_{ib}^{b}$ and ${\text{ω}}_{ib}^{b}$, then we have:

(30) $$\left[\begin{array}{ccc} {\dot{f}}_{\text{ibx}}^{b}\left(t_{1}\right) & {\dot{f}}_{\text{iby}}^{b}\left(t_{1}\right) & {\dot{f}}_{\text{ibz}}^{b}\left(t_{1}\right)\\ {\dot{f}}_{\text{ibx}}^{b}\left(t_{2}\right) & {\dot{f}}_{\text{iby}}^{b}\left(t_{2}\right) & {\dot{f}}_{\text{ibz}}^{b}\left(t_{2}\right)\\ {\dot{f}}_{\text{ibx}}^{b}\left(t_{3}\right) & {\dot{f}}_{\text{iby}}^{b}\left(t_{3}\right) & {\dot{f}}_{\text{ibz}}^{b}\left(t_{3}\right) \end{array}\right]\;\left[\begin{array}{c} G_{11}\\ G_{12}\\ G_{13} \end{array}\right]=\left[\begin{array}{c} {\dot{\omega }}_{\text{ibx}}^{b}\left(t_{1}\right)\\ {\dot{\omega }}_{\text{iby}}^{b}\left(t_{2}\right)\\ {\dot{\omega }}_{\text{ibz}}^{b}\left(t_{3}\right) \end{array}\right]$$

Let:

(31) $$\begin{array}{c} \text{A}={\left[\begin{array}{ccc} {\dot{\text{f}}}_{ib}^{b}\left(t_{1}\right) & {\dot{\text{f}}}_{ib}^{b}\left(t_{2}\right) & {\dot{\text{f}}}_{ib}^{b}\left(t_{3}\right) \end{array}\right]}^{T}\;\\ B={\left[\begin{array}{ccc} {\dot{\omega }}_{ib}^{b}\left(t_{1}\right) & {\dot{\omega }}_{ib}^{b}\left(t_{2}\right) & {\dot{\omega }}_{ib}^{b}\left(t_{3}\right) \end{array}\right]}^{T} \end{array}$$

Suppose that during the process of flight, there are two straight line paths with three different times, making rank ***A*** = 3. If the rank of matrix ***A*** is three, then ***A*** has the nonzero determinant. According to Cramer's rule, the Equation (30) has a unique solution. In other words, *G*_2_ can be uniquely determined. Following the derivation above, the *G*_2_ and *G*_3_ also can be uniquely determined in the same condition. Hence the g-sensitivity coefficients matrix with nine elements is observable. That is, the gyro g-sensitivity errors are known. Since all the terms in Equation (9) except ***b****_a_* are known, ***b****_a_* can be uniquely determined.

The foregoing results are summarized by the following conclusion. For the integrated MEMS INS/GPS system with gyro g-sensitivity errors, if the aircraft flies along a trajectory on which there are more than one path segments that ${\dot{\text{C}}}_{b}^{e}=0$, and meeting the following conditions on those segments:

(a) one path segment on which there are two linearly independent ${\dot{\text{f}}}_{ib}^{b}$
(b) when *G* is a three-element diagonal matrix, more than one path segments on which each element of ${\dot{\text{C}}}_{b}^{e}=0$ exist non-zero time;
(c) when *G* is a nine-element matrix, two path segments on which there are three different time, making rank ***A*** = 3;

Then the position, velocity, body attitude, gyro and accelerometer bias, and gyro g-sensitivity coefficients are all observable.

The conclusion only gives a sufficient condition in that system may be observable under other situations. The path segment that ${\dot{\text{f}}}_{ib}^{b}$ requires the aircraft hold a constant attitude, which is easily satisfied when the aircraft flies straight. The influence of weather during the fight is ignored. The first condition requires that the direction of ${\text{f}}_{ib}^{b}$ should change on a straight path segment. For the aircraft, it can be fulfilled at the instantaneous moment that the aircraft begins to turn after flying along a straight line with varying acceleration. Since this moment is instantaneous, we predict that the observability of ***b****_a_* and ***b****_s_* is relatively weak and that this type of maneuver needs to be operated repetitiously to achieve a satisfactory estimation. Because the rank of diagonal matrix *G* is three, the second conditions are relatively easy to achieve. For example, suppose that the aircraft flies along a straight line with varying acceleration and turning at times with constant attitude. For the remaining conditions, if the aircraft keeps on climbing straight after a steady turn with varying accelerations, it also can be required. In brief, when the maneuvering of the aircraft consists of varying accelerated linear motions, steady turns and climbing, the g-sensitivity coefficients are observable. Additionally, since the benefit of estimating the nine states and estimating only three states is similar in a practical application [6], we just given the observability condition for the nine-element of g-sensitivity coefficient matrix, and conditions (a) and (b) are validated by simulations.

## 4. Simulation and Results

**Figure 1.**
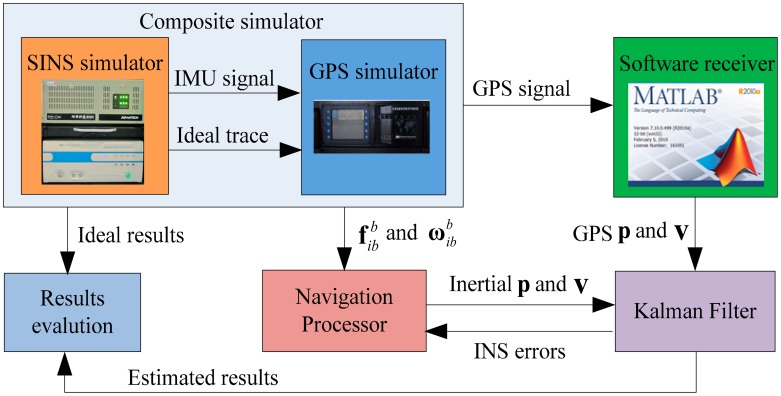
The test system composition based on composite simulator.

To support the theoretical discussion above, some simulations are carried out. The error states vector is composed of position, velocity, attitude, gyro and accelerometer bias, and g-sensitivity coefficients. It is evident that a state is observed, and then it can be estimated. It is well known that an unobservable state cannot be estimated, even in the most favorable situation [18,19]. The test system is illustrated in Figure 1. We edit the parameters of aircraft movements based on the condition to the composite simulator, and it can provide the actual satellite signal and synchronous inertial measurement data. After that, the GPS signal is tracked in a Matlab-based software receiver, and the inertial measurement data is calculated, respectively. The Kalman filter was chosen as data fusion to estimate the states. To test the global observability conditions (a) and (b), we compared the estimated results with the ideal results provided by the composite simulator.

The update rate of the INS is 200 Hz, and the navigation solution is corrected by the Kalman filter using position and velocity measurements from a single-antenna GPS receiver at the rate of 100 Hz. Assuming all the noises in process and sensor to be Gaussian white. The initial attitude errors of roll, yaw, and pitch are supposed 5°. Suppose that the standard deviation (STD) of position measured by GPS receiver is 5 m, and the STD of velocity is 0.5 m/s. The statistics of MEMS sensor noises in simulation are presented as follows:

(1) The constant bias of the gyro: 0.014 °/s;
(2) The gyro random noises: 0.0028 °/s;
(3) The gyro g-sensitivity coefficient: 10 °/h/g;
(4) The constant bias of the accelerometer: 0.01 m/s^2^;
(5) The accelerometer random noises: 0.001 m/s^2^.

**Figure 2.**
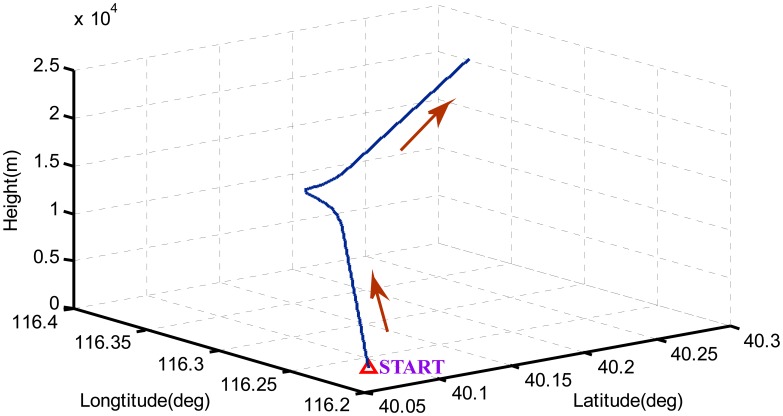
The 3-D trajectory of the aircraft.

**Figure 3.**
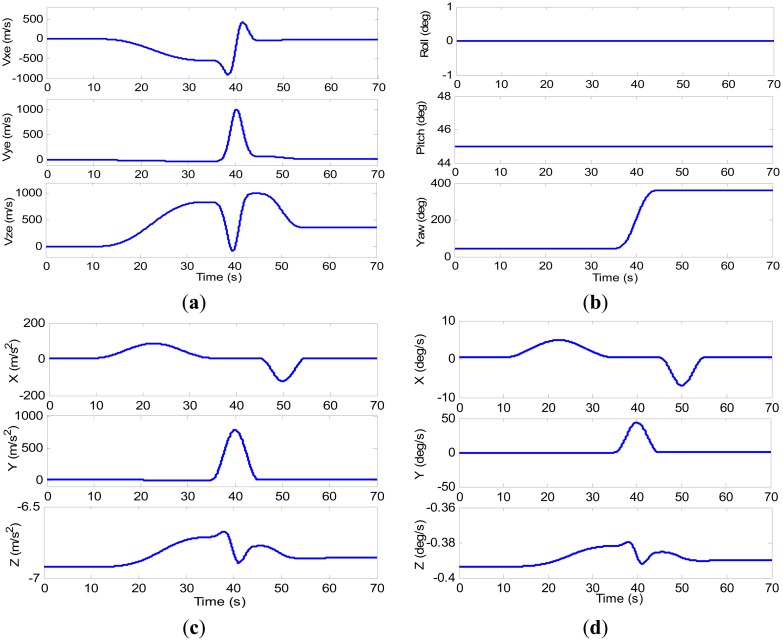
(**a**) Velocity of the aircraft in earth-centered earth-fixed (ECEF); (**b**) Attitude of the aircraft; (**c**) Specific force of the aircraft in b frame; and (**d**) G-sensitivity errors of the gyro.

According to the global observability conditions, we design a flight trajectory including varying accelerated linear motions, steady turns and climbing. The influence of weather during the fight is ignored and allows our focus on the observability of error states. In the first 10 s, the aircraft stands still. Then, it accelerates straight for 25 s with varying accelerations, keeps a constant velocity and turns right for 10 s. After that the aircraft decelerates with varying values for 10 s, and then ascends until the end. The kinematics of the aircraft are shown in Figures 2 and 3.

The g-sensitivity errors of gyro are shown in Figure 3d. The values of specific force are shown in Figure 3c and the three g-sensitivity coefficients mentioned above are the same constant, so those errors can be calculated by the Equation (16). Since the velocity was relatively high, this could cause a certain centripetal acceleration at high values, and the peak specific force of the right axis is about 80 g, as shown in Figure 3c.

**Figure 4.**
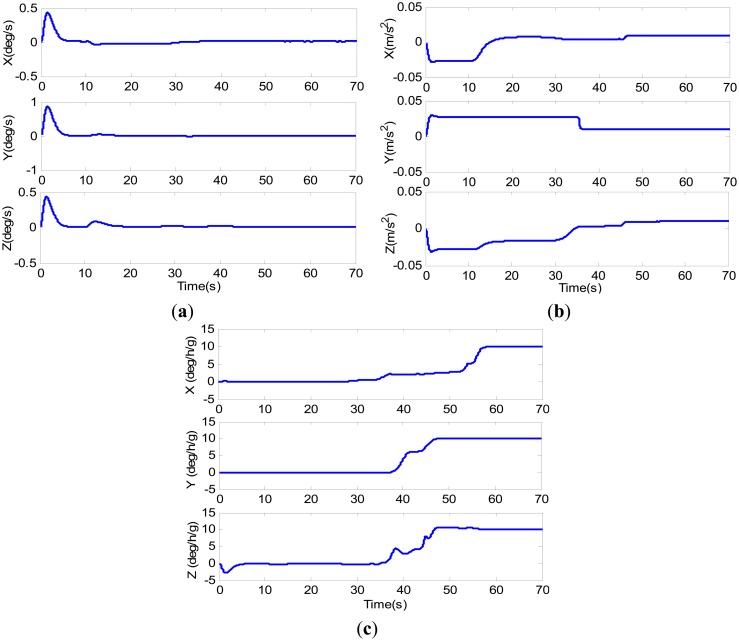
(**a**) Estimation of the gyro bias; (**b**) Estimation of the accelerometer bias; and (**c**) Estimation of the gyro g-sensitivity coefficients.

As show in Figure 4a,b, the estimated bias of gyro and accelerometer converge toward their true value, respectively, after 10 s. As the aircraft begins to fly in a rectilinear fashion accelerating non-uniformly, the bias of gyro and that of accelerometer in the forward and down directions evidently converged. Referring to Figure 3a, we can find that the direction of ${\dot{\text{f}}}_{ib}^{b}$ sharply changed at the begin-to-turn moment, so that ${\dot{\text{f}}}_{ib}^{b}$ is linearly independent on this moment. Due to the modeling errors, the estimations converge but do not approach the true values. After the aircraft fly two maneuvering, the estimated values have come very close to the truth in 50 s. The estimations of g-sensitivity coefficients are shown in Figure 4c. The g-sensitivity coefficients drift in the first 10 s when the aircraft remains stationary, and begin to converge when the aircraft starts accelerating. As the aircraft is climbing and turning in the 35–45 s, the the g-sensitivity coefficients converge toward their true value. Referring to Figure 3a, we can find that the specific force change in all three directions in in 45–50 s, which is sufficient for the requirements for the g-sensitivity coefficients to be observable. After the aircraft passes the second curve segment, the estimated values have come very close to the truth. The statistical errors are showed in Table 1. The average estimated values mean that the estimated values are average when they have been converged to their true value nearly.

**Figure 5.**
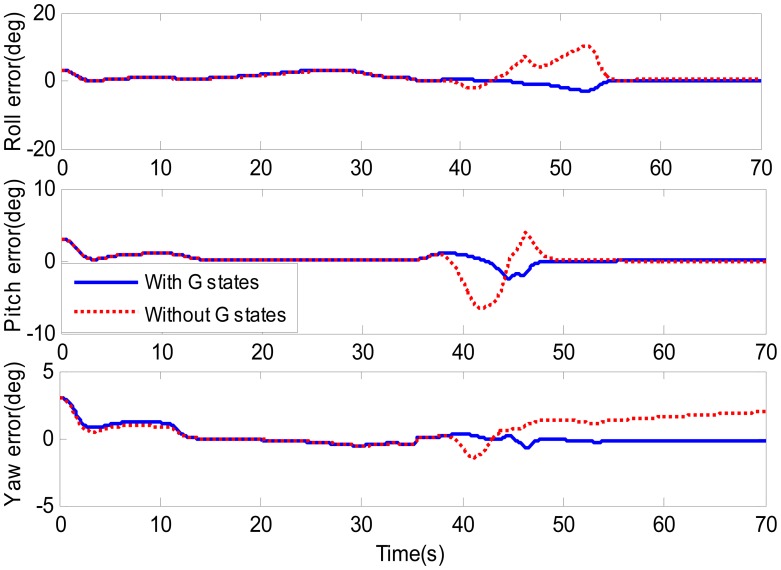
Estimation of the attitude errors.

To evaluate the attitude accuracy of integration system after the g-sensitivity coefficients are correctly estimated, another simulation was carried out with the Kalman filter without g-sensitivity error states. According to Equations (13)–(15), the g-sensitivity errors mainly affect the accuracy of attitude estimation, and had no effect on position and velocity. Their estimation accuracy mainly depends on the measurement. Therefore, the estimations of position and velocity are not shown in the article. The simulation conditions and the maneuvering were the same as indicated above. The simulation results are given in Figure 5 and Table 2.

In Figure 5, the g-sensitivity coefficients almost do not change (as shown in Figure 4c), so the attitude errors showed the same changing trend in 0–35 s. At the begin-to-turn, the observable requirements for g-sensitivity coefficients are sufficient, so their values in all directions come to coverage. By considering G-states, the attitude error begin to converge, and their values have come very close to zero (see blue lines) until 55 s. Referring to Figure 4c, we can find that the g-sensitivity coefficients have also come to their truth in all three directions. After 55 s, all three attitude errors are close to zero (as shown in blue lines). But for the filter without G-state, the attitude error could not be converging (see red dashed lines in Figure 4) owing to the g-sensitivity errors. In 55–70 s, the g-sensitivity errors in the *x*-direction and *y*-direction are close to zero (as shown in Figure 3d), so the roll and pitch errors without G-states showed the same changing with considering g- sensitivity errors. Noticing that, the yaw error is always not converging until the end, which is owed to the *z*-direction g-sensitivity error. From Table 2, we can easily find that the attitude accuracy is obviously improved.

## 5. Conclusions

In this paper, we have studied the observability of errors in an integrated MEMS INS/GPS system, in which we mainly focus on the gyroscope g-sensitivity errors. Two different types of Kalman filter were established, which could be used to estimate the g-sensitivity coefficients by adding them as states to the filter. The observability of the nonlinear system describing the integration system was investigated based on the global observability analysis approach. A sufficient condition for the global observability of error states that includes position, velocity, attitude, gyro and accelerometer bias, and g-sensitivity coefficients was presented. Aiming at two different kinds of g-sensitivity coefficient matrices, three-element and nine-element, the relationship between their observability and the aircraft movements was revealed. To verify the theoretical results, a numerical simulation based on the composite simulator was carried out. The results showed that all the estimated states could be made observable by maneuvering based on the conditions. Through compensating the g-sensitivity errors, the attitude accuracy of the integration system has been improved remarkably.

## Figures and Tables

**Table 1.** Comparison between the truth values and the average estimated values.

**Estimated States**	**Bias of Gyroscopes (°/h)**			**Bias of Accelerometer (mg)**			**G-sensitivity Coefficients (°/h/g)**		

	**Forward**	**Right**	**Down**	**Forward**	**Right**	**Down**	***G*_11_**	***G*_22_**	***G*_33_**
Truth values	50	50	50	1	1	1	10	10	10
Average estimated values	49.621	49.910	50.414	0.993	0.997	0.986	9.955	9.986	9.969

**Table 2.** Comparison between gyro g-sensitivity errors before compensation and after compensation on estimation of attitude errors.

**Attitude errors**	**Roll error(°) RMS (1σ)**	**Pitch error (°) RMS (1σ)**	**Yaw error (°) RMS (1σ)**
Before compensation	2.25	1.23	1.34
After compensation	0.54	0.35	0.17
